# Plasma Zinc Level in Hepatitis C Patients With or Without Beta Thalassemia Major; Is There Any Difference?

**DOI:** 10.5812/hepatmon.11138

**Published:** 2013-08-12

**Authors:** Mohammad Abbasinazari, Bita Behnava, Yunes Panahi, Azita Hajhossein Talasaz, Shima Salimi, Maryam Keshvari, Leila Mehrnoush, Pegah Karimi Elizee, Mohammad Gholami Fesharaki, Mansour Asgharinia, Seyed Moayed Alavian

**Affiliations:** 1 Department of Clinical Pharmacy, Shahid Beheshti University of Medical Sciences, Tehran, IR Iran; 2 Baqiyatallah Research Center for Gastroenterology and Liver Disease, Baqiyatallah University of Medical Sciences, Tehran, IR Iran; 3 Chemical Injuries Research Center, Baqiyatallah University of Medical Sciences, Tehran, IR Iran; 4 Department of Clinical Pharmacy, School of Pharmacy, Tehran University of Medical Sciences, Tehran, IR Iran; 5 Iranian Blood Transfusion Organization Reaserch Center, Tehran, IR Iran; 6 Middle East Liver Disease, Tehran, IR Iran

**Keywords:** Zinc, Beta-Thalassemia, Hepatitis C, Iran

## Abstract

**Background:**

Zinc deficiency has been reported frequently in hepatitis C patients in the literature. Furthermore, a decrease in zinc level has been shown in beta thalassemia major as well. Iranians consume a large amount of phytate in their regimens which can bind with zinc and decrease its gastrointestinal absorption.

**Objectives:**

This study was designed to determine plasma zinc level in an Iranian sample with the diagnosis of hepatitis C with or without concomitant beta thalassemia major.

**Patients and Methods:**

Between April 2011 and April 2012, plasma zinc level was determined via atomic absorption method, in 130 hepatitis C patients with or without beta thalassemia major in a known referral center of hepatic diseases in Tehran, Iran.

**Results:**

Mean ± standard deviation (SD) of plasma zinc levels was determined as 0.78 ± 0.22 mg/L. Also zinc level was 0.76 ± 0.19 mg/L and 0.80 ± 0.24 mg/L in thalassemic and non thalassemic patients, respectively. T-test analysis showed that there is no significant difference between these two groups regarding plasma zinc level (P = 0.235).

**Conclusions:**

It is concluded that zinc level of studied patients is less than which is reported in normal Iranian population. Moreover, there is not a significant difference in plasma zinc levels between thalassemic and non thalassemic patients and it seems to be a common problem in both ones. Addition of zinc supplement may be recommended in both groups in order to optimize the nutritional support and probably improve the treatment response.

## 1. Background

Zinc is one of the most important micronutrients in humanbeings. It can be found in all parts of the body with 85% of the total body zinc in musculoskeletal system (1). Zinc is essential for activation of about 300 different enzymes in vivo and is regarded to be necessary for the metabolism (2). Previous studies showed that patients with chronic hepatitis C have low plasma zinc level (3, 4). Increased urine depletion, poor nutrition and decreased absorption of zinc from gastrointestinal tract have been counted for the reduction of zinc level in hepatitis C patients (3, 5).

There is a high rate of thalassemia in Iran as it is located in the median of the thalassemia belt (6). Despite of progress which is made in the screening of blood derivatives, patients with beta thalassemia major are at high risk of hepatitis C because of the blood transfusion from infected donors (7, 8). It has been reported that patients with beta thalassemia major have zinc deficiency (9). It is suggested that iron chelators such as desferrioxamine which is essential for the treatment of beta thalassemia major increases urinary zinc depletion (10).

## 2. Objectives

The aim of the present study is to determine the mean plasma level of zinc in two groups of hepatitis C patients; those with concomitant beta thalassemia major and those without beta thalassemia major and to determine if there is any difference in zinc levels between these groups.

## 3. Patients and Methods

The present study was conducted on hepatitis C patients (either with beta thalassemia major or without beta thalassemia major). Patients were selected from a well-known referral center for the treatment of hepatitis C in Tehran, Iran (Baqiatallah Research Center for Gastroenterology and Liver Diseases, Hepatitis Tehran Clinic). Ethical committee approval was obtained before starting the study on the provision of Helsinki declaration (2000). Also all the patients had given informed consent. Patients were excluded from the study if they had taken any product containing zinc supplement during the last month or during the study. All of the patients had a HCV RNA level of more than 50 U/mL six months prior the study. Also all were positive on the basis of PCR for hepatitis C. Diagnosis of beta thalassemia major was based on peripheral blood smear examination and hemoglobin electrophoresis (Hb F > 20%) of the patients from early years of life. After selecting the patients, blood samples were obtained from the patients arm. Blood samples were centrifuged in 5000 rpm for 7 minutes and plasma was separated. Remained plasma samples were stored at the -80 °C. We used atomic absorption method for assessment of zinc level in the patients as the usual method for zinc evaluation (11). Concentrations of 0.1, 0.3, 0.5 and 0.7 ppm of zinc sulfate were prepared as standards. Atomic absorptions of them were determined for obtaining the standard curve. After ending the sample gathering, plasma zinc levels were evaluated by using atomic absorption (Perkin Elmer 1100 B).

## 4. Results

During our study period, 130 patients with the diagnosis of Hepatitis C were included in one year (April 2011 to April 2012). Of them, 80 were hepatitis C patients with beta thalassemia major and 50 were only hepatitis C patients.

Age, sex and the presence of cirrhosis in selected patients were shown in Table 1. Analysis of chi-square showed that there is not any difference between the sex of patients in two groups (P = 0.278). Also there is a significant difference in the distribution of age between two groups (P < 0.001). With regards to cirrhosis, there is not any difference between two groups (P = 0.710) 

. The mean plasma zinc level was 0.78 ± 0.22 mg/mL in the selected patients. In patients with diagnosis of hepatitis C and beta thalassemia major, plasma zinc level was 0.76 ± 0.19 mg/L. Meanwhile, in non thalassemic patients with diagnosis of hepatitis C plasma zinc level was 0.80 ± 0.24 mg/L. Analysis of t-test showed that there is not any significant difference between the two regarding to plasma zinc level (P = 0.235). Also, the mean plasma level of zinc was 0.76 ± 0.21 mg/mL in cirrhotic patents versus 0.80 ± 0.23 mg/mL in non-cirrhotic patients. Analysis of t-test showed that there is not any significant difference between the plasma level of zinc in these two groups level (P = 0.436).

**Table 1.** Demographic Data of Studied Patients

	Thalassemic patients	Non thalassemic patients	P value
**Mean Age, y, Mean ± SD**	26.10 ± 5.08	31.94 ± 7.27	< 0.001
**Sex, Female/Male, No. **	37/43	18/32	0.278
**Presence of cirrhosis, Yes/No**	42/38	29/21	0.710

## 5. Discussion

Yuasa et al have reported that zinc may play an important role as a negative regulator of HCV replication in the genome length of HCV RNA replicating cells (12). Plasma level of zinc is often decreased in chronic hepatitis C patients (13, 14). Moreover, zinc deficiency has been reported frequently in patients with beta thalassemia major as well. For the first time, chronic zinc deficiency in patients with beta thalassemia major has been reported by Cavdar and Arcasoy from Turkey (15). Chelation of zinc with chelator agents such as deferoxamine may be an important cause of zinc deficiency in the thalassemic patients (10). As a result, the determination of zinc level in patients with both thalassemia major and hepatitis C is recommended. In this study we determined plasma zinc level in two groups of hepatitis C patients; those who had beta thalassemia major simultaneously and those without thalassemia. Our data revealed that the mean plasma zinc level in both groups (0.78 ± 0.22 mg/mL) is less than what has been reported in Iranian healthy volunteers (0.89 ± 0.16mg/mL) (16). Nevertheless, there was not any significant difference between the mean plasma level of zinc between hepatitis C patients with or without thalassemia (P = 0.235). The distribution of sex and the presence of cirrhosis was not significantly different between these two groups (P = 0.278 and P = 0.710, respectively). Although the mean age of both groups was in the same range, statistical analysis showed a significant difference between the two (P < 0.001). This can be introduced as a limitation of our study. Further studies are needed with matched ages in two groups.

The low plasma zinc level is common in patients with liver cirrhosis due to decreased intake, absorption, bioavailability, and also malabsorption. Protein synthesis is also reduced in the liver of cirrhotic patients. Therefore, there would be a deficiency in the synthesis of metallothionein as an important zinc binding protein (formed by liver) involved in the zinc homeostasis (17). Whereas mechanisms of zinc deficiency in hepatitis C patients are more known in cirrhotic patients, zinc concentration is reduced in both cirrhotic and chronic hepatitis compared with control subjects (18). In this study we have not seen any difference between the plasma level of zinc in cirrhotic versus non-cirrhotic patients (P = 0.436).

Nutritional status plays an important role in zinc concentration. For example despite the beneficial effects of whole wheat breads, they consist of high amounts of phytic acid which is believed to negatively decrease the absorption of zinc (19) . Because Iranian people usually consume large amount of phytate in their regimen, low zinc concentration has been reported frequently in this population (20).

Overall this study showed a decreased zinc level in hepatitis C patients either with beta thalassemia major or non-beta thalassemia major. Therefore, addition of zinc to the therapeutic regimen of hepatitis C patients with or without beta thalassemia major can be recommended.
